# Response to Hulman and colleagues regarding “Glucotypes reveal new patterns of glucose dysregulation”

**DOI:** 10.1371/journal.pbio.3001092

**Published:** 2021-03-11

**Authors:** Alessandra Breschi, Dalia Perelman, Michael Paul Snyder

**Affiliations:** Stanford University, Department of Genetics, Stanford, California, United States of America; Duke University, UNITED STATES

## Abstract

In a response to a Formal Comment critiquing their model for classifying individualized glucose patterns into glucotypes, these authors stand by their results and conclusions, which can be reproduced using their publicly available data, and maintain that improved algorithms for analyzing CGM data will continue to emerge and enrich the field.

We thank Hulman and colleagues for their comment on our article “Glucotypes reveal new patterns of glucose dysregulation,” and we are pleased that we have the opportunity to respond. We have the following comments:

First, our study [1] was one of the first to systematically analyze continuous glucose monitoring (CGM) data from healthy individuals and people with prediabetes (as well as several with type 2 diabetes), and the discovery that many individuals that were classified as healthy or with prediabetes were exhibiting moderate to severe glucose excursion, i.e., “spikes” was surprising to many, including us. From this finding, it was reasonable to develop quantitative measures that would take into account mean and glucose variability.

As such, we explicitly say in our manuscript that glucotypes reflect both mean and glycemic variability. We wrote: “*We note that using CGM*, *the classes*, *from low to severe*, *increase in both variability and mean glucose concentration*, *making them a more comprehensive metric of glycemic state compared to existing measurements*.” To us, this was logical as it fits with the concept that excursions outside of range should be incorporated into a glucose health metric (Fig 1 panels b and c in [1]).

**Fig 1:**
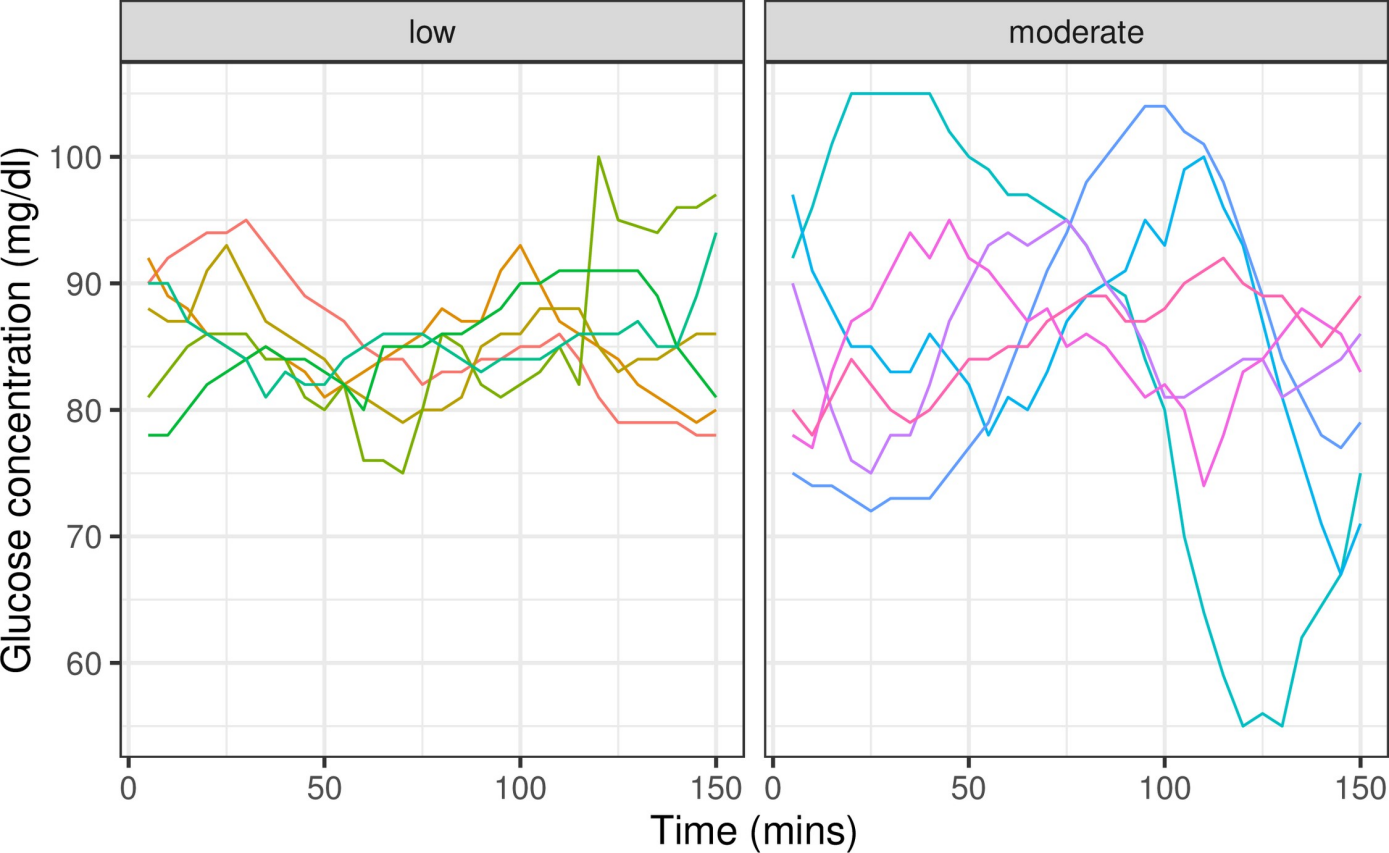
Examples of time windows with the same mean glucose concentration, but different glucotype—low to moderate classification. While there is a correlation between mean glucose concentration and standard deviation, such that glycemic profiles with higher glucose concentration are also more variable, we show examples of time windows where the mean glucose concentration is comparable, but with different glycemic variation. In such cases, the glucotype algorithm by Hall and colleagues, which uses the overall shape of the time window, captures both mean glucose concentration and variation. The windows are selected from S3 Data from our study [1]. All selected windows have mean glucose concentration between 85 and 86 mg/dl. While this concentration is too low for a severe glucotype classification, the windows are classified as low or moderate reflecting their glycemic variability.

Second, Hulman and colleagues state that the glucotype mostly just captures the mean. However, we note that the mean and standard deviation of glucose variation are correlated, but only partially (R = 0.45), and thus the mean does not capture all of the useful information from a CGM. Indeed, Supporting information Fig A of Hulman and colleagues shows that the glucotypes are quite mixed for certain mean glucose levels, and examples of people with the same mean but different glucotypes are shown in Figs 1 and 2 below. Thus, the fact that we came up with a method that captures more than the mean is novel and, we believe, valuable. We also built a web-based App, and many readers have found it to be quite useful.

**Fig 2:**
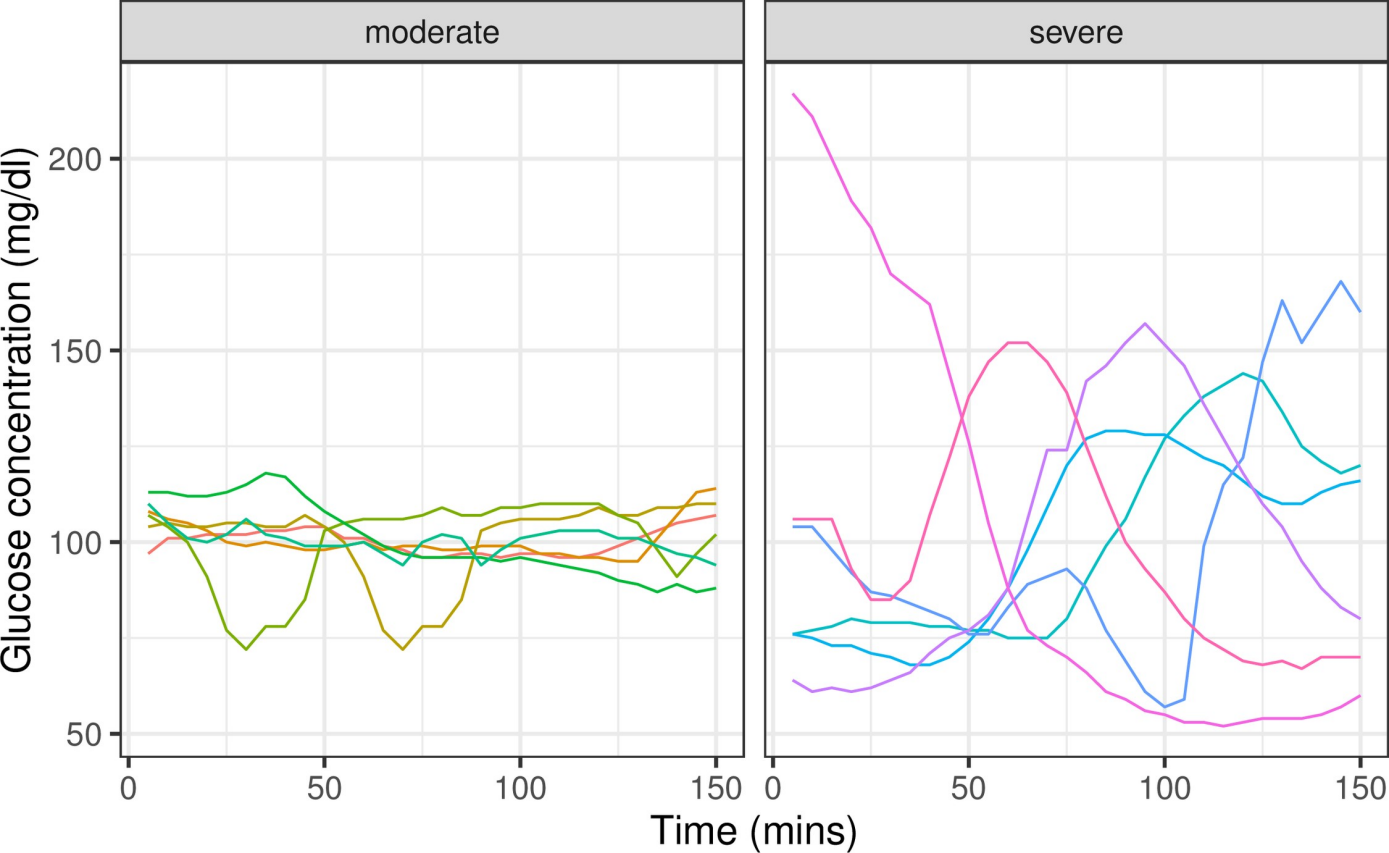
Examples of time windows with the same mean glucose concentration, but different glucotype—moderate to severe classification. All selected windows have mean glucose concentration between 100 and 101 mg/dl. While this concentration is too high for a low glucotype classification, the windows are classified as moderate or severe reflecting their glycemic variability.

Third, we agree that there is no clear separation between the glucotype clusters; glucose dysregulation metrics are usually continuous (e.g., Hemoglobin A1c), and we do have numerical values. However, as this is a new metric with its own scale and not familiar to most users or health care providers, providing categories facilitates the health message; this is similar to using categories like “healthy,” “prediabetes,” and “diabetes.” We suggested a novel approach to classify individuals through a clustering algorithm that systematically groups people into 3 categories, beyond setting a cutoff based on mean glucose values. Moreover, we can categorize phenotypes at the windows level which many people found valuable. Note that for the users who prefer a more nuanced metric, our web-based App does provide a numeric value corresponding to the glucotype.

Fourth, our study was primarily based on a mostly healthy and prediabetic population, and our glucotype measure was reasonable for this group—indeed, it is the population we were most focused on. As noted by the authors, the Maastricht study is enriched with people with diabetes and older, and the PRE-D Trial study was for overweight and obese people with prediabetes. Thus, that many of their participants are in the severe category is not surprising, as noted by the authors. Similarly, we expect the results to be different in different populations (healthy versus diabetic, etc.), and those populations with more glucose dysregulation will exhibit more severe glucotypes.

Fifth, since our publication more than 2 years ago, we do expect there will be new ways to analyze CGM data for classification of glucose dysregulation. We and many others are working on this. Others are working on composite measures, which are not new ways of analyzing the data, but ways of combining summary variables from CMG data [2].

Finally, and perhaps importantly, we are one of few groups (and the only one thus far for type 2 diabetes), who made their CGM data freely available, and it is easily downloadable as a zipped text file from PLOS website. The published data from the Maastricht study and PRE-D studies must be requested with a specific use request. While the PRE-D group appears eager to work with us, the Maastricht study requires an application with a lot of requested information, including research intent, funding, revision of potentially deriving publications, and other information. Even providing this information does not guarantee access to the data and, if successful, requires both a collaboration and manuscript approval. In fact, in our email request for access to their data, we were discouraged from applying. We hope that in the future, all data and analysis code are made freely available.

In summary, we stand by our results and conclusions which we feel are valuable. They can be reproduced with our publically available data. We have no doubt improved algorithms for analyzing CGM data will continue to emerge and enrich the field.
